# Lifetime Obesity in Patients with Eating Disorders: Increasing Prevalence, Clinical and Personality Correlates

**DOI:** 10.1002/erv.2166

**Published:** 2012-03-02

**Authors:** Cynthia Villarejo, Fernando Fernández-Aranda, Susana Jiménez-Murcia, Eva Peñas-Lledó, Roser Granero, Eva Penelo, Francisco J Tinahones, Carolina Sancho, Nuria Vilarrasa, Mónica Montserrat-Gil de Bernabé, Felipe F Casanueva, Jose Manuel Fernández-Real, Gema Frühbeck, Rafael De la Torre, Janet Treasure, Cristina Botella, José Manuel Menchón

**Affiliations:** 1 Department of Psychiatry, University Hospital of Bellvitge—IDIBELLBarcelona, Spain; 2 CIBER Fisiopatología Obesidad y Nutrición (CIBERObn), Instituto Salud Carlos IIISpain; 3 Clinical Sciences Department, University of BarcelonaSpain; 4 University of ExtremaduraBadajoz, Spain; 5 Laboratori d'Estadística Aplicada, Departament de Psicobiologia i Metodologia, Universitat Autònoma de BarcelonaSpain; 6 Department of Diabetes, Endocrinology and Nutrition, Hospital Clínico Universitario Virgen de VictoriaMálaga, Spain; 7 Endocrinology and Nutrition Department, Bellvitge Universitary Hospital—IDIBELLBarcelona, Spain; 8 Dietetics Unit, Bellvitge Universitary HospitalBarcelona, Spain; 9 Division of Endocrinology, Complejo Hospitalario Universitario de Santiago de CompostelaSpain; 10 Department of Diabetes, Endocrinology and Nutrition, Institut d'Investigació Biomèdica de Girona (IdlBGi) Hospital Dr Josep TruetaGirona, Spain; 11 Department of Endocrinology and Nutrition, University of NavarraPamplona, Spain; 12 Human Pharmacology and Clinical Neurosciences Research Group, Neuroscience Research Program, IMIM—Hospital del Mar Research InstituteBarcelona, Spain; 13 Institute of Psychiatry, Psychological Medicine, Section of Eating Disorders, King's College LondonLondon, UK; 14 University Jaume ICastellón, Spain; 15 CIBER, Salud Mental (CIBERSAM), Instituto Carlos IIISpain

**Keywords:** obesity, eating disorders, personality, psychopathology

## Abstract

**Objectives:**

: The aims of our study were to examine the lifetime prevalence of obesity rate in eating disorders (ED) subtypes and to examine whether there have been temporal changes among the last 10 years and to explore clinical differences between ED with and without lifetime obesity.

**Methods:**

: Participants were 1383 ED female patients (DSM-IV criteria) consecutively admitted, between 2001 and 2010, to Bellvitge University Hospital. They were assessed by means of the Eating Disorders Inventory-2, the Symptom Checklist-90—Revised, the Bulimic Investigatory Test Edinburgh and the Temperament and Character Inventory—Revised.

**Results:**

: The prevalence of lifetime obesity in ED cases was 28.8% (ranging from 5% in anorexia nervosa to 87% in binge-eating disorders). Over the last 10 years, there has been a threefold increase in lifetime obesity in ED patients (*p* < .001). People with an ED and obesity had higher levels of childhood and family obesity (*p* < .001), a later age of onset and longer ED duration; and had higher levels of eating, general and personality symptomatology.

**Conclusions:**

: Over the last 10 years, the prevalence of obesity associated with disorders characterized by the presence of binge episodes, namely bulimic disorders, is increasing, and this is linked with greater clinical severity and a poorer prognosis. Copyright © 2012 John Wiley & Sons, Ltd and Eating Disorders Association.

## Introduction

Eating disorders (ED) and obesity share some biological and environmental risk factors (Bachar, Gur, Canetti, Berry, & Stein, 2010; Bulik, Sullivan, & Kendler, 2003; Haines, Kleinman, Rifas-Shiman, Field, & Austin, 2010; Root et al., 2011), behaviours (Gunnard et al., 2011; Roemmich, Lambiase, Lobarinas, & Balantekin, 2011) and intermediate neurocognitive phenotypes (Danner, Ouwehand, van Haastert, Hornsveld, & de Ridder, 2012; Van den Eynde & Treasure, 2009; Volkow, Wang, Fowler, Tomasi, & Baler, 2011). A controversial theory postulates that ED and obesity form part of a broad spectrum of eating-related and weight-related disorders (Marcus & Wildes, 2009; Volkow & O'Brien, 2007; Wilson, 2010). Nevertheless, contradictory findings in the literature do not allow differentiating ED according to weight-related phenotypes.

Obese patients with a comorbid ED [mainly binge-eating disorder (BED)] have higher eating (Fassino, Leombruni, Piero, Abbate-Daga, & Giacomo Rovera, 2003; Hsu et al., 2002), general (Bulik, Sullivan, & Kendler, 2002; Fandiño et al., 2010; Zeeck, Stelzer, Linster, Joos, & Hartmann, 2011) and personality psychopathology (Nasser, Gluck, & Geliebter, 2004) and a poorer prognosis (Hsu et al., 1998). However, it remains unclear the rate of lifetime obesity across ED diagnostic subtypes and their associated phenotypical features, as well as their stability overtime.

The aims of this study were to examine the prevalence and distribution of lifetime obesity across the range of ED patients to examine temporal changes from 2001 to 2010 and to evaluate the existence of clinical differences between subjects with an ED with and without obesity.

## Methods

### Participants

The participants were 1383 female ED patients [261 anorexia nervosa (AN), 551 bulimia nervosa (BN), 448 not otherwise specified ED (EDNOS), and 123 binge-eating disorders (BED)] with a mean age of 27.0 years (*SD* = 8.25). Patients were consecutively admitted to the ED Unit of our Psychiatry Department, between 2001 and 2010, and diagnosed according to DSM-IV-TR criteria (APA, 2000), by means of Structured Clinical Interview for DSM IV Axis I Disorders (First, Gibbon, Spitzer, & Williams, 1996). Experienced psychologists and psychiatrists completed the clinical assessment during two structured face-to face interviews. These covered lifetime presence of obesity as well as additional information related to clinical questions such as age of onset, duration, course of the disorder, minimum and maximum body mass index (BMI), presence of family history of obesity and childhood obesity (defined as positive when a subject recalled ever been diagnosed with obesity by a physician in childhood). Patients with lifetime obesity are defined in this work as those having childhood obesity and/or a BMI greater than or equal to 30 kg/m^2^ in adulthood. To determine current obesity, the interviewer directly measured weight and height during this session to calculate BMI.

We obtained written informed consent from all participants, and the Ethics Committee of our hospital approved the study.

### Assessment

We developed a comprehensive battery of assessments to quantify ED symptoms, general psychopathology and personality. The battery included the Eating Disorders Inventory-2 (Garner, 1991), the Bulimic Investigatory Test Edinburgh (Henderson & Freeman, 1987), the Symptom Checklist-Revised-90—Revised (Derogatis, 1990) and the Temperament and Character Inventory—Revised (Cloninger, 1987). All these questionnaires are validated in Spanish and have been described previously (Derogatis, 2002; Garner, 1998; Gutierrez et al., 2001; Rivas, Bernabé, & Jiménez, 2004).

### Statistical analysis

Statistical analysis was carried out using spss19 for Windows (IBM Corporation, Armonk, NY). The prevalence of lifetime and childhood obesity was estimated for each diagnostic subtype Clinical and personality differences between ED patients with and without lifetime obesity were analysed using analysis of variance adjusted by age and duration of the ED as covariates.

**Figure 1:**
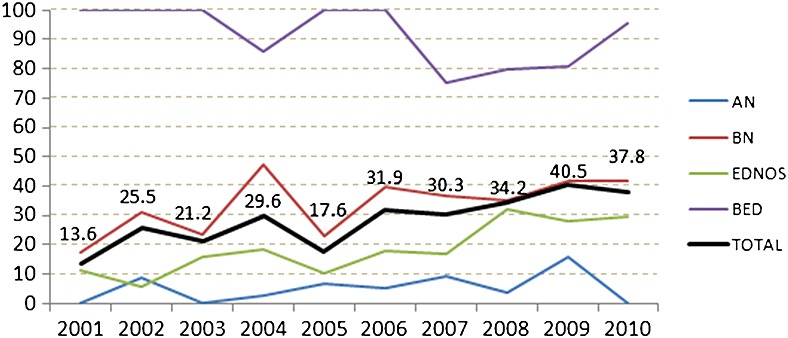
Percentage of lifetime obesity overtime, between 2001 and 2010, in consecutive ED referrals (*N* = 1383). *Note: labeled % of the total ED /year*

## Results

### Lifetime obesity rate across eating disorders diagnostic subtypes and temporal effects

Three hundred and ninety eight ED patients had lifetime obesity (28.8%, 95% CI: 26.4 to 31.2). BED individuals had the highest rate (87.8%; 95% CI: 78.6 to 95.7) followed by BN (33.2%, 95% CI: 29.4 to 37.2) and EDNOS (21.2%, 95% CI 17.7; 25.2%) with AN showing the lowest rates (4.6%; 95% CI: 2.64; 7.86).

There was a similar pattern of distribution for family history of obesity (62.8% BED, 33.8% BN, 26.3% EDNOS, 13.3% AN; *p* < .001) and childhood obesity (28.9% BED, 13% BN, 12.2% EDNOS, 3.6% AN; (*p* < .001).

In addition, a positive linear trend in the prevalence of lifetime (*p* < .0001) (Figure 1) and childhood obesity (*p* < .0001) and current BMI (*p* < .0001) was found over the duration of the clinical collection (Figures in supplementary data).

### Clinical, psychopathological and personality differences between eating disorders with and without lifetime obesity

Participants with ED and lifetime obesity had higher minimum and maximum BMI, a later age of onset and longer ED duration (Table 1), higher eating severity scores (*p* < .001; by means of EDI-2, Bulimic Investigatory Test Edinburgh and bingeing frequency) and greater general psychopathology (*p* < .005; by means of Symptom Checklist-90—Revised) when compared with ED patients without lifetime obesity. They also showed higher harm avoidance and lower traits on persistence, self-directedness and cooperativeness than ED without this condition.

**Table 1:** Comparison of clinical, psychopathological and personality measures across eating disorders patients with and without lifetime obesity (ED + OB versus ED − OB)

	ED − OB (*N* = 985)		ED + OB (*N* = 398)		ANOVA			
			
	Mean	SD	Mean	SD	*p*	MD	95% CI for MD	
BMI: minimum	17.6	2.5	22.0	4.3	**<.0001**	3.656	3.268	4.044
BMI: maximum	23.4	2.9	35.0	7.1	**<.0001**	10.117	9.544	10.690
Age of onset (years)	18.7	5.2	22.0	9.6	**<.0001**	3.237	2.428	4.046
Duration of ED (years)	6.4	5.6	9.4	7.8	**<.0001**	3.041	2.289	3.794
Weekly binges	3.5	5.9	5.6	6.3	**<.0001**	1.961	1.159	2.763
Weekly vomits	4.6	7.4	4.1	7.0	.956	0.045	−0.916	1.006
Weekly laxatives	3.3	13.0	3.8	12.3	.956	0.612	−1.078	2.301
Weekly diuretics	1.1	4.8	2.1	6.9	.065	0.801	0.065	1.536
BITE: symptoms	18.7	7.8	23.0	5.6	**<.001**	3.424	2.220	4.628
BITE: severity	10.3	7.4	12.9	7.3	**<.001**	2.307	1.097	3.517
EDI: drive for thinness	12.7	6.8	14.2	5.2	.**002**	1.392	0.559	2.225
EDI: body dissatisfaction	14.7	8.2	20.1	6.7	**<.001**	5.032	4.013	6.051
EDI: interceptive awareness	10.6	6.6	12.4	6.7	**<.001**	1.871	0.993	2.749
EDI: bulimia	5.9	5.6	9.14	5.3	**<.001**	2.989	2.255	3.724
EDI: interpersonal distrust	5.6	4.6	6.2	4.7	.**012**	0.809	0.202	1.415
EDI: inefficacy	10.3	7.1	12.0	7.0	**<.001**	1.810	0.880	2.740
EDI: maturity fears	8.0	5.7	8.2	5.6	.266	0.457	−0.289	1.203
EDI: perfectionism	5.6	4.3	5.5	4.5	.987	−0.005	−0.576	.567
EDI: impulsivity	6.5	5.8	7.5	6.4	**<.001**	1.576	0.805	2.348
EDI: ascetic	6.6	4.4	7.8	4.0	**<.001**	1.186	0.624	1.748
EDI: social insecurity	7.5	5.0	8.2	4.7	.**005**	0.947	0.298	1.597
EDI: total score	94.0	44.3	111.2	40.3	**<.001**	17.985	12.32	23.66
SCL: somatization	1.7	0.9	2.0	0.9	**<.001**	0.324	0.199	.448
SCL: obsessive–compulsive	1.9	0.9	2.1	0.9	.**007**	0.168	0.050	.286
SCL: interpersonal	1.9	0.9	2.2	0.9	**<.001**	0.280	0.159	.401
SCL: depressive	2.2	0.9	2.4	0.9	.**003**	0.191	0.072	.310
SCL: anxiety	1.7	0.9	1.9	0.9	.**042**	0.128	0.005	.251
SCL: hostility	1.4	1.0	1.5	1.1	.**008**	0.185	0.051	.319
SCL: phobic anxiety	1.0	0.9	1.2	1.0	.**008**	0.177	0.049	.304
SCL: paranoid	1.4	0.9	1.6	0.9	.**002**	0.202	0.086	.319
SCL: psychotic	1.3	0.8	1.5	0.8	**<.001**	0.224	0.120	.327
SCL: GSI	1.7	0.8	1.9	0.8	**<.001**	0.221	0.120	.322
SCL: PST	63.8	18.9	67.2	17.1	.**003**	3.892	1.457	6.326
SCL: PSDI	2.3	0.6	2.5	0.6	**<.001**	0.185	0.109	.262
TCI: novelty seeking	102.4	16.4	102.6	16.1	.289	1.349	−0.791	3.488
TCI: harm avoidance	115.4	20.0	121.1	17.9	.**003**	4.381	1.809	6.952
TCI: reward dependence	103.6	15.4	103.9	17.0	.638	−0.596	−2.710	1.518
TCI: persistence	111.1	21.5	106.8	21.9	.**030**	−3.496	−6.371	−.622
TCI: self-directedness	118.4	21.8	112.3	21.1	.**000**	−5.231	−8.082	−2.379
TCI: cooperativeness	135.7	17.3	133.1	17.7	.**023**	−3.013	−5.311	−.715
TCI: self-transcendence	65.5	14.8	65.9	15.2	.901	0.125	−1.844	2.093

Results obtained in analysis of variance (ANOVA) adjusted by age, duration of ED and subtype. *p* values include Bonferroni–Holm's correction. In bold: *p* < .05; ED, eating disorders; OB, obesity; BMI, body mass index; BITE, Bulimic Investigatory Test Edinburgh; EDI, Eating Disorders Inventory; SCL, Symptom Checklist-90; GSI, Global Severity Index; PST, Positive Symptom Total; PSDI, Positive Symptom Disease Index; TCI, Temperament and Character Inventory.

## Discussion

The main finding was the high and increasing temporal prevalence of lifetime obesity in ED (28.8% of cases), particularly in BED/BN.

The second main finding of our study was that ED patients with lifetime obesity were characterized by later age of onset, higher ED severity and greater general psychopathology when compared with patients without lifetime obesity. This group had a poorer prognosis (Bulik, Sullivan, Joyce, Carter, & McIntosh, 1998; Fairburn et al., 1995). As reported in previous studies, people with obesity history had a stronger family history of obesity (Nuñez-Navarro et al., 2011; Whitaker, Jarvis, Beeken, Boniface, & Wardle, 2010) with increased childhood obesity (Brisbois, Farmer, & McCargar, 2011). This combination is associated with higher levels of eating and general psychopathology (Dingemans & van Furth, 2012) and more dysfunctional personality traits (namely higher harm avoidance, lower persistence, self-directedness and cooperativeness; Nasser et al., 2004).

The third main finding was that the threefold increase in obesity in the ED population, mainly in BED and BN, over the last 10 years, is much higher than the changing prevalence recorded in women in general population surveys (FESNAD-SEEDO, 2011; OECD, 2010). This is in agreement with the observation previously reported in other ED-related populations (McAlpine et al., 2010; Müller et al., 2012).

The results of this study should be considered within the context of the following limitations: First, the retrospective and self-report data collection procedures may limit the validity and the reliability of our findings. Second, the cross-sectional design does not allow us to determine the causality of the variables assessed. Therefore, it will be important to confirm the relevance of these results by longitudinal research that will determine the patterns of temporal association.

Research suggests that adolescents may simultaneously experience multiple weight-related problems, increasing severity over time. Therefore, clinicians treating individuals with obesity or ED should remain vigilant for the emergence of additional weight-related problems. Further, in the treatment of patients with ED and obesity, multidisciplinary approaches addressing both conditions may eventually produce superior effectiveness than those that concentrate exclusively on the problem for which the individual sought treatment.

In summary, lifetime obesity seems to become more and more prevalent among ED patients (as preceding, coexisting or consequent condition). Therefore, ED services may need to adjust their treatments to match the changing clinical profile and also when designing early or tertiary prevention strategies (Gonzalez, Penelo, Gutierrez, & Raich, 2011; Koskina et al., 2011).
