# Supplementary material for: Lifetime Obesity in Patients with Eating Disorders: Increasing Prevalence, Clinical and Personality Correlates
Source: Eur Eat Disord Rev. 2012 Mar 2;20(3):250–4. doi: 10.1002/erv.2166 (PMC3510304; doi:10.1002/erv.2166)
Supplement: Supplementary file 1 [file erv0020-0250-SD1.pdf]

Table 2-online

Clinical and sociodemografic features among ED subtypes

		**AN** (N=261)	**BN** (N=551)	**EDNOS** (N=448)	**BED** (N=123)	**Total** (N=1383)
Age (years); *mean (SD)*		24.78 (7.13)	27.29 (7.74)	25.85 (7.76)	34.63 (9.84)	27.00 (8.25)
Studies level; *%*	Primary	29.4%	34.5%	29.6%	34.5%	31.9%
	Secondary	52.5%	51.0%	56.6%	47.8%	52.9%
	University	18.0%	14.5%	13.7%	17.7%	15.2%
Num. of treatments; *mean (SD)*		1.14 (1.54)	0.70 (0.99)	0.77 (1.06)	0.41 (0.64)	0.78 (1.13)
Age of onset of ED; *mean (SD)*		19.15 (5.22)	19.50 (6.90)	18.88 (5.68)	24.41 (11.25)	19.66 (6.91)
Duration of ED; *mean (SD)*		5.40 (5.07)	7.86 (6.09)	6.70 (6.32)	10.42 (9.02)	7.25 (6.43)
BMI; *mean (SD)*		16.02 (1.43)	24.48 (5.80)	21.14 (4.48)	35.52 (6.12)	22.78 (6.96)
BMI minimum; *mean (SD)*		15.16 (1.47)	19.71 (3.05)	18.29 (2.81)	24.04 (4.37)	18.79 (3.69)
BMI maximum; *mean (SD)*		21.80 (2.91)	27.93 (6.48)	25.23 (5.47)	37.29 (6.58)	26.72 (6.88)
Family history of obesity; *%*		13.3%	33.8%	26.3%	62.8%	30.2%

AN: Anorexia nervosa. BN: Bulimia nervosa. EDNOS: Eating disorder not otherwise specified. BED: Binge eating disorder. SD: standard deviation. BMI: body mass index (kg/m^2).

Table 3-online

Prevalence (with the 95% confidence interval, exact estimation) of lifetime obesity and obesity during childhood stratified by diagnosis subtype

	AN (N=261)		BN (N=551)		EDNOS (N=448)		BED (N=123)
**Obesity: life-time**; prevalence in %	4.6 (2.64; 7.86)		33.2 (29.4; 37.2)		21.2 (17.7; 25.2)		87.8 (80.9; 92.5)
Comparison between subtypes:	AN vs BN		0.097 (0.05; 0.18)*		BN vs EDNOS		1.848 (1.39; 2.46)*
OR coefficients	AN vs EDNOS		0.179 (0.10; 0.33)*		BN vs BED		0.069 (0.039; 0.122)*
	AN vs BED		0.007 (0.003; 0.02)*		EDNOS vs BED		0.037 (0.021; 0.067)*
	AN (N=267)		BN (N=569)		EDNOS (N=459)		BED (N=124)
**Obesity: childhood**; prevalence in %	3.6 (1.89; 6.65)		13.0 (10.4; 16.1)		12.2 (9.44; 15.6)		28.9 (21.6; 37.6)
Comparison between subtypes:	AN vs BN		0.250 (0.12; 0.51)*		BN vs EDNOS		1.076 (0.74; 1.58)
OR coefficients	AN vs EDNOS		0.269 (0.13; 0.56)*		BN vs BED		0.367 (0.23; 0.59)*
	AN vs BED		0.092 (0.04; 0.20)*		EDNOS vs BED		0.341 (0. 21; 0.56)*

*AN: Anorexia nervosa. BN: Bulimia nervosa. EDNOS: Eating disorder not otherwise specified. BED: Binge eating disorder. *Significant OR coefficient.*

Figure 2-online

Prevalence of childhood obesity (percentage, %)

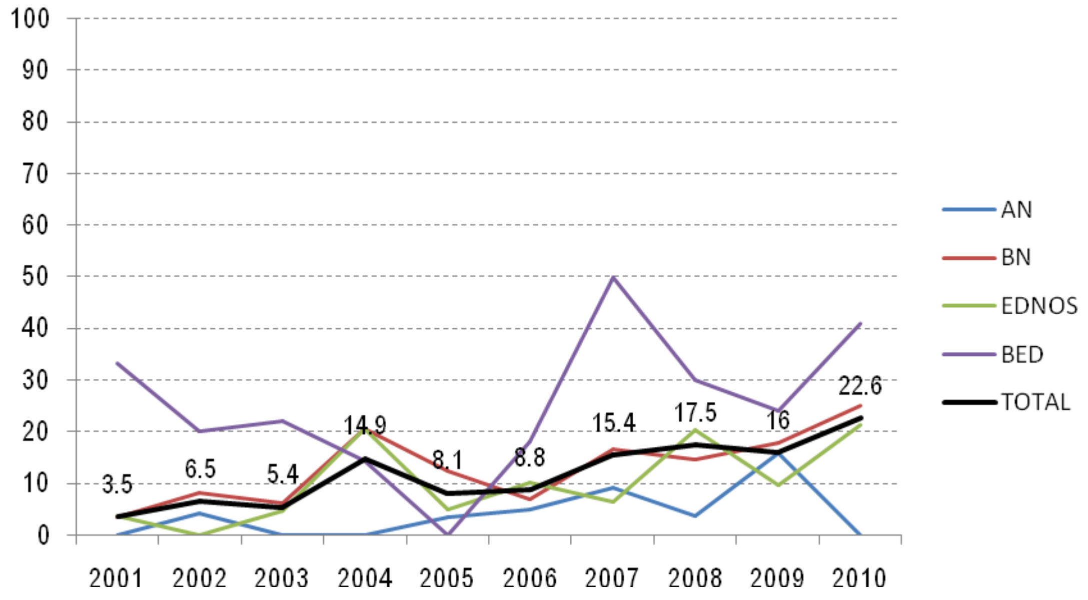

Note: labeled % of the total ED /year

Figure 3-online

Current Body Mass Index (mean values) among the groups

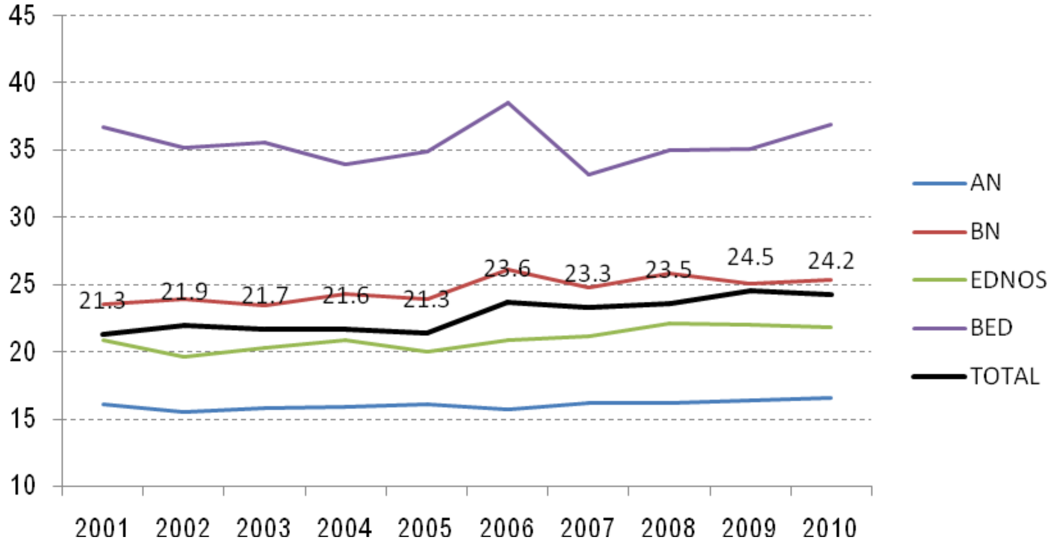

Note: labeled % of the total ED /year
