# Supplementary figures and images for: Lifetime Obesity in Patients with Eating Disorders: Increasing Prevalence, Clinical and Personality Correlates
Source: Eur Eat Disord Rev. 2012 Mar 2;20(3):250–4. doi: 10.1002/erv.2166 (PMC3510304; doi:10.1002/erv.2166)

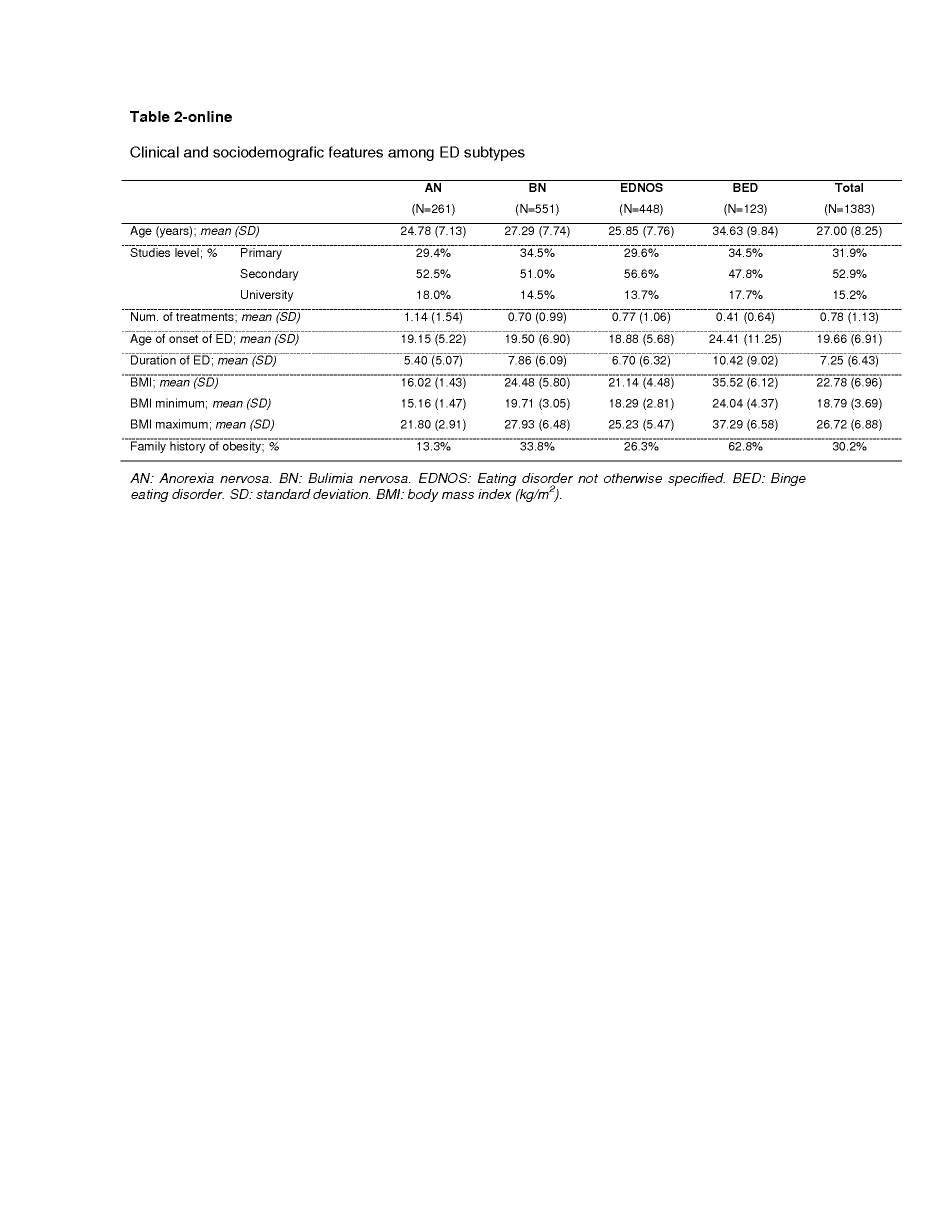

Supplement: Supplementary file 2 [file erv0020-0250-SD2.png]
